# Deconstructing age(s): an analysis of the different conceptions of age as a legal criterion for access to assisted reproductive technologies

**DOI:** 10.1093/jlb/lsac036

**Published:** 2022-12-15

**Authors:** Andrea Martani, Eva De Clercq, Christian De Geyter, Guido Pennings, Tenzin Wangmo, Bernice Simone Elger

**Affiliations:** Institute for Biomedical Ethics, University of Basel, Basel, Switzerland; Institute for Biomedical Ethics, University of Basel, Basel, Switzerland; Reproductive Medicine and Gynaecological Endocrinology (RME), University Hospital, University of Basel, Basel, Switzerland; Department of Philosophy and Moral Science, Bioethics Institute Ghent (BIG), Ghent University, Gent, Belgium; Institute for Biomedical Ethics, University of Basel, Basel, Switzerland; Institute for Biomedical Ethics, University of Basel, Basel, Switzerland; University Center of Legal Medicine, University of Geneva, Switzerland

**Keywords:** Access to medically Assisted Reproduction - Advanced Parental Age, Assisted Reproductive Technologies, Comparative Legal Analysis, Ethics in Reproduction, Reproductive Health Law

## Abstract

Whether there should be restrictions for access to Assisted Reproductive Technologies (ART) is a matter of continuous medical, societal, and ethico-legal debate. One of the most controversial topics in this context is the use of parental age as a criterion to limit access to ART. Views are divided on whether there should be an upper age limit for one or both parents and on where such limits should be. Although this debate is centered around the issue of ‘age’ and although *age*-related limits are present in many legislations, the intrinsic ambiguity of the term `age' is largely overlooked.

In this article, we build on gerontological, medical, and sociological literature on the concepts of ‘age’ and ‘aging’ to distinguish three conceptions of age that are relevant for ART regulation: the chronological, the biological, and the social-cultural one. Beyond mapping out these conceptions of age, we describe how they relate to ART and reproduction, and illustrate the advantages and disadvantages of relying on each of them as a basis for limiting ART access. Finally, we propose a template for defining legal age limits for ART access in the law, based on the refined understanding of the different conceptions of age that we outline and we discuss two potential objections to our proposal.

## I. INTRODUCTION

Assisted Reproductive Technologies (ART) have become a globally widespread treatment modality for infertile couples during the past few decades. The term ART refers to a number of different procedures aimed at solving fertility problems of individuals by performing a series of events involved in human reproduction (from fertilization to the development of an embryo) in a medical laboratory.^1^ Amongst the most common procedures there are: (i) in vitro fertilization (IVF), where—after the retrieval of oocytes either from the woman trying to obtain a pregnancy (homologous IVF) or from a donor (heterologous IVF)—‘the oocyte is inseminated with a number of washed spermatozoa allowing fertilization under conditions similar to those in the ampulla of the Fallopian tubes’; and (ii) intracytoplasmic sperm injection, where ‘with the help of a micropipette one single, viable spermatozoon is inserted directly into the […] cytoplasm [of the oocyte]’.^2^ A recent study focusing on several European countries showed that in 2017 the annual number of children born with the support of ART reached almost 200,000, corresponding to >3 percent of the total registered births. In some countries (eg, Denmark or Spain), the proportion of children conceived through ART is even higher (5.6 and 7.9 percent, respectively).^3^ This might be due to their reimbursement policies for fertility treatments, which are considered particularly supportive, according to the recently published European Atlas of Fertility Treatment Policies.^4^ In contrast, there are also countries where regulations on ART access and financing are much more restrictive, for example by limiting access to (heterosexual) couples only, or by limiting the coverage through social health insurance to a specific number of treatment cycles.^5^ This diversity in regulation of ART is the result of medical, ethical, and legal debates on whether there should be limits for accessing ART and on what the nature of these limits should be.

One of the most fiercely debated aspects of ART regulation concerns the role that parental age should have as a criterion to access such technologies. At the macro-level, it is discussed whether there are any prospective parents who are simply too old to potentially have a child by getting access to ART. At a more detailed level, the discussion on the role of parental age is further differentiated depending on the specific type of ART in question (eg, heterologous vs. homologous IVF) or the specific type of access in question (eg, publicly funded ART vs. ART paid out-of-pocket).^6^ The law plays a central role in this respect, since several countries (at least in the Global North) have decided to enshrine considerations about parental age into legislation on ART access, either in terms of general eligibility or for coverage of ART through public health care.^7^ Even in the absence of legislation, there are often guidelines or other soft law instruments which refer to the role that parental age should play in access to ART. For example, a report of the Swedish National Council on Medical Ethics acknowledges that there are no statutory upper age limits for IVF in the country, but that county councils usually enforce upper limits of 37–41 years and it argues that anyway at least ‘one parent must be young enough to be able to take responsibility for the child until he or she reaches adulthood’.^8^ In the US—one of the most notable exceptions where there are no age limits in hard law—the Ethics Committee of the American Society for Reproductive Medicine has explicitly discouraged to provide ‘donor oocytes or embryos to women over 55 years of age, even when they have no underlying medical problems’.^9^ Since age is often referenced in laws or guidelines as an important variable to be considered when deciding whether to grant access to (or cover the expenses for) ART, policymakers need to choose how age limits should be articulated, ie, which age cutoffs are appropriate.^10^

The dilemmas of which age cutoff for prospective parents should be determined in ART regulation stem mainly from two—partially interconnected—reasons. First, scientific evidence about the negative effects of advanced parental age on offspring is not decisive. Although it is acknowledged that advanced parental (maternal^11^ and paternal^12^) age may increase the health risks for the mother and the child, there is also evidence that having older parents has some beneficial effects for the long-term health and well-being of the child (eg see Kieron and Mikko and David et al.^13^^,^^14^). Second, normative arguments on whether it is ‘right’ or not to grant access to ART beyond a certain age are in dissonance as well, as highlighted by reviews on the ethical aspects of paternal and maternal advanced age.^15^^,^^16^ Proponents of more stringent age limits often rely on argumentations based on the idea that limits should be posed so that technology does not tamper with what is considered ‘natural’ reproduction.^17^ Other proponents of stricter age limits justify their conviction on paternalistic grounds, eg, age limits are necessary to protect women from unnecessary medical interventions (and thus physical and mental harm), given the low chances of obtaining and sustaining a viable pregnancy after a certain time. Those in favor of more flexible age limits (or no age limits at all), on the contrary, often base their arguments on the principle of reproductive autonomy, or they highlight the risk of discrimination, for example that age limits might turn into a form of ageism – ie, the expression of ‘prejudice by one age group towards other age-groups’.^18^ Overall, the question of where exactly should age limits be posed remains a contentious issue, as shown by the substantially divergent legal rules concerning age limits in different countries.^19^

Arguably, one of the reasons why there is much disagreement on whether and which age limits should be enshrined in legal provisions on ART access is that age in itself is an ambivalent and multifaceted concept, whose exact meaning is hardly ever addressed. This is particularly surprising, since ‘aging is arguably the most familiar yet least well-understood aspect of human biology’.^20^ Indeed, in many scientific fields (eg, sociology, psychology, gerontology), it is common knowledge that the term *age* can refer to extremely different conceptions of aging and time (eg see James and Cunningham^21^). Legal scholars, on the contrary, usually discuss age using a chronological definition,^22^ but at the same time—especially when it comes to ethically loaded topics such as ART—they tend to often conflate chronological, biological, and socio-cultural considerations. The importance of differentiating between various conceptions of legal age at a general level has recently been highlighted by Boni-Saenz.^23^ In this article, we pick up on his recommendation to ‘think more deeply about the large landscape of age-based law that regulates our lives’ and we discuss the meaning of parental age in relation to the regulation of ART access (in the Global North). More specifically, our aims are: (a) to map out the different conceptions of age which underlie age limits in legal rules on ART access; (b) to illustrate the advantages and disadvantages of relying on each of these conceptions as a basis for limiting ART access; and (c) to propose a suitable template for defining legal age limits for ART access, based on the refined understanding of the different conceptions of age that we outline.

In the next sections, we draw on gerontological, medical, and sociological literature on ‘age’ and ‘aging’ to identify three different conceptions of age which are most relevant for the debate about parental age limits to ART access: chronological (II.A), biological (II.B), and socio-cultural age (II.C). After outlining the main features of each of these conceptions, we demonstrate how these features interplay with the ethical debate on setting upper age limits for parents who wish to conceive a child through ART (II.A.1, II.B.1, and II.c.1). For each of the conceptions of age, we also provide examples of laws and policies that embody them (II.A.2, II.B.2, and II.C.2) and we discuss the advantages and disadvantages of having upper age limits that are based on a chronological, biological, or socio-cultural conception of age (II.A.3, II.B.3, and II.C.3). We finally propose a template of how rules on parental age limits to ART could be defined, and tackle two potential objections to our proposal (III).

## II. CONCEPTIONS OF AGE AND ART

Before we proceed we want to make two preliminary remarks. First, it is important to acknowledge that any conception of age—including the three which we focus on—is to some extent socio-politically constructed and thus embodies normative considerations. Chronological age as, for example, presupposes a conception of time as a linear, quantified, standardized, and universalized process, which is entrenched into capitalistic ideology.^24^ Biological age and—needless to say—socio-cultural age are also underpinned by normative and political considerations: the former because biomedical knowledge on aging is usually produced within specific normative frameworks,^25^ ie, normative considerations concerning declining fertility rates or appropriate time for childbearing might bear on what is conveyed as a matter of fact about biological aging; the latter because it is based explicitly on what members of society believe to be the ‘right’ timing for different life stages.

Second, it is crucial to remember that the different conceptions of age are never entirely independent of each other, especially when operationalized or implemented in legal norms or any other form of regulation. This is due to the fact that, for example, the biological and the socio-cultural conceptualizations of age are often commingled, since ‘in gerontology […], we have no [terms] to distinguish between biological and social ageing’, a vocabulary gap which ‘makes it very easy to think of biological and social ageing as the same thing’.^26^ This becomes particularly evident in the discourse around the concept of a biological clock, as discussed below in II.B.3. Or else, the interconnection between different conceptions of age is evident when considering the justification behind chronological age limits. Indeed, although legal rules usually delineate age limits in chronological terms and legal scholars—when they analyze age—generally assume a chronological definition,^27^ it should not be overlooked that there might be socio-cultural or biological motivations behind the choice of a specific chronological cutoff. The problems related to using chronological age as a variable in legal rules, whilst at the same time grounding the choice of the selected cutoff (or age-limit) in biological or socio-cultural motivations are further explored in II.A.3.

Despite their embeddedness in normative frameworks and their interconnectedness, it is still important to differentiate between different conceptions of age. Indeed, if a system of law decides to impose age limits on access to ART (or indeed anything else), it is only by understanding the exact nature of such limits that it is possible to evaluate their appropriateness. As Boni-Saenz underscored,^28^ the stakes for defining what legal age generally means in a system of law are already high. They are even higher when a certain conception of age is used specifically to delineate the limits for accessing an important medical service like ART.

### II.A. Chronological Age

The most direct way how age can be conceived as a criterion to determine access to ART is through its chronological dimension. This means considering the aging process (from birth onwards) as something determined by calendar-time, regardless of the subjective situation of the person going through that process, her perceptions, or those of the society where she lives.^29^ In brief, the chronological age of any person can be defined as ‘the lapsed time in days, months and years since birth’.^30^ Chronological age is essentially based on an understanding of time as an absolute and linear movement, against which the process of aging can be measured in an objective (ie, each measurement of age based on this conception of time will lead to the same result) and unequivocal fashion (ie, regardless of their lived experiences, two people born on the same day will be considered chronologically equally old). According to this understanding of time, the basic formula to calculate chronological age is quite obvious, since it is—as formalized by Schaie: chronological age = time of measurement (eg, 2022)—birth cohort (eg, 1982).^31^ Chronological age possesses an almost intrinsic and intuitive appeal due to its straightforwardness and its presumed objectivity. Its value is often taken for granted in the field of scientific gerontology and beyond, since it is assumed that

‘Chronological age is one of the most useful single items of information about an individual if not the most useful. From this knowledge alone an amazingly large number of general statements or predictions can be made about his anatomy, physiology, psychology and social behavior’.^32^

The appeal of the chronological conception of age has also determined its common use throughout different sectors of societal life, to the extent that ‘chronological age [controls] many aspects of education, work and retirement’.^33^ Arguably, the most striking example of the relevant role that chronological age plays is the case of adulthood: across most western societies, the age of 18 has been chosen as a chronological marker to determine the passage to adulthood. At this exact moment, all people reach the age of majority, as far as the law is concerned. In the blink of one day (the 18th birthday), the same persons can proceed to marry, to carry full legal responsibility for their actions, to manage their own property, and exercise fully a series of other civil rights (eg, voting).

#### II.A.1. Chronological age and ART access

As outlined, chronological age is widely used to organize the setup and functioning of society, and ART makes no exception. Indeed, the literature discussing the question of when *old* is *too old* for ART access reveals that such debate often emphasizes the chronological meaning of age. For example, in an article by Bewley and colleagues, the authors reflect on the necessity to better inform women about reproduction and (in)fertility, and on the limits of ART as a measure to achieve pregnancy at a later age. Although their discussion concentrates primarily on the influence of biological factors on fertility and on healthy pregnancy, they then summarize their main message by suggesting that there is a defined chronological timespan where it is best to have children. Indeed, their subtitle reads that ‘the most secure age for childbearing remains 20-35’ and they warn against the risks of ART with women older than such age.^34^

An emphasis on chronological age transpires also from an article by Caplan and Patrizio.^35^ In presenting their argument in favor of ‘restrictions on […] the age of women eligible to use infertility services’, the authors start their discussion positing that the birth numbers of mothers older than 37 years have steadily increased in recent years.^36^ They list seven old mothers giving birth when they were between 65 and 70 years and underline the various complications that these pregnancies and deliveries characterized. Although the authors then reflect on rather biological aspects of aging, they nevertheless emphasize that mere chronological factors are important: in fact, they claim that one question at the center of the ethical dilemma of ART access is: ‘How should a woman’s age and life expectancy factor into a clinic policy concerning access to services?’.^37^

Similarly, also medical practitioners underscore the importance of chronological age to limit access to ART. For instance, in a recent qualitative study with medical professionals providing ART services in the United States, some practitioners admitted relying principally on a chronological understanding of age to decide if prospective parents should get access to ART.^38^ One physician reported that in their clinic the chronological age of prospective mothers can be decisive:

they [prospective mothers] must be no older than 54 years. If they are >50 years, they cannot have any medical problems. Therefore, you can have medical problems and be 49 years, we will still do the transfer, but if you have any medical problems then you are ruled out at 50 years.^39^

Two other providers claimed that the cumulative chronological age of the prospective parents must be below a certain sum for them to get access to ART. More specifically, they argued that patients cannot ask for ART if the sum of the age of the (prospective) parents adds up to anything above 80, 90, 100, or 110 (depending on the clinic). In another study by Friese, one interviewee reported having gone to a clinic where they refused to let a couple undergo ART because ‘they used a rule of thumb that if the combined age of the parents was over one hundred, they wouldn’t do a donor egg procedure’.^40^

#### II.A.2. Chronological age-limits for ART access in the law

Having parental age limits for accessing ART based on a chronological conception of age is arguably the most widespread regulatory solution across the countries where ART are regulated. A recent survey of ART legislation across Europe showed that the majority of the countries analyzed have a chronological age limit for prospective mothers, ranging between 45 and 50 years.^41^ For example, in Belgium, prospective mothers must be below 45 years of age to request access to ART, and the implantation of the embryo (or insemination) cannot occur after they turn 48.^42^^,^^43^ The same study showed that European legislations normally refrain from setting age limits for prospective fathers. When they do, the limits that they pose reflect essentially a chronological conception of age. In Sweden, prospective fathers cannot be older than 56, and in Finland and Portugal the limit is 60 years.^44^ Swiss legislation stands out in that art. 3 para 2 lit. b requires that both prospective parents have an age that will likely allow them to care for their child until it reaches the age of majority.^45^ Although related to life expectancy, also this age limit is predominantly chronological, since it refers to the relationship between the chronological age of the parents and that of the prospective children.^46^

#### II.A.3. Advantages and disadvantages of chronological age-limits

Having explicit chronological age limits to regulate access to ART has some advantages. First, it is not ambiguous and it facilitates decisions dealing with family planning, since limits are the same for all (prospective) parents: the latter are made aware that there is a specific and precise moment in their life from which they categorically will not be able to have access to ART anymore. Second, an exclusively chronological age limit is helpful for medical professionals who provide ART services. In the United States, where there is no regulatory chronological age cut-off for ART, healthcare personnel have highlighted that they often take decisions about parental age limits and ART based on gut feelings or perceptions of public opinion (ie, *their* perception of whether the public would consider a certain age as ‘too old’—which might even differ from what the public actually thinks—see also socio-cultural age below).^47^ Having a clear chronological age cutoff beyond which treatment is not allowed, might thus avoid arbitrary decisions based on the views of the treating medical team resulting in unconscious biases. Third, legal age limits of a chronological nature are always easy to use and enforce. In his discussion on appropriate age cutoffs for social security benefits, Persad highlighted that verifying if someone is 65 years of age is probably the simplest of assessments, thus saving also many costs.^48^

On the other side, an exclusive reliance on chronological age has also several disadvantages. To begin with, although chronological age is easily measurable, quantifiable, and comparable, purely chronological considerations cannot provide us *by themselves* with any valuable criterion for when to put age limits. Chronological age limits are thus somehow empty shells: their justifications are normally based on criteria of a biological or socio-cultural nature. Indeed, chronological cutoffs are not used because chronological age has a meaning in itself, but since it ‘serves as a good proxy for a variety of social and biological variables of interest’.^49^ This can be problematic because also bio-medical knowledge in itself does not provide an unequivocal numerical (in form of years) cut-off where boundaries should be drawn. As Smajdor explains: ‘If there were an [chronological] age at which the risks of IVF rise dramatically, things might be easier. But this is not the case. Risks rise incrementally’.^50^ So, deciding on a chronological cutoff based on biological considerations might be difficult to justify. For example, in Germany for women covered by social health insurance, reimbursement of ART is limited until the age of 40.^51^ When a person contested this limit in a trial, the court held that such a limit is justified due to the fact that data from the German IVF-Register show that chances of a clinical pregnancy in women at the age of 40 are half compared with those in women aged 30.^52^ But it is not clear why this comparison between success rates at the age of 30 and 40 would provide a meaningful reason to stop reimbursement *exactly* at 40. The same report^53^ shows that there is actually little difference in the success rates between women aged 40 and those aged 41, so it is not clear why the latter should be excluded from ART access. Moreover, more recent data from the same register^54^ show that chances of clinical pregnancy in women above 40 have been increasing, thus raising the question why the law has not caught up. In short, hidden behind these chronological age limits, there are justifications based on biological considerations about the chances of a clinical pregnancy, which are in turn debatable.^55^ Chronological age limits might hide not only biological, but also implicit socio-cultural considerations about age and reproduction. For example, it is not clear why in Belgium the legislator picked *exactly* the age of 47 (with no exceptions/flexibility) as the last year in which women can demand the implantation of an embryo as part of ART. The choice of this exclusively chronological age-cutoff seems to be only ‘the result of a mix of prejudice, a vague theory on reproductive time span, paternalism and concerns about safety and health of women’.^56^

Second, chronological age is not necessarily an accurate measure for the specific context of ART. In their discussion on whether (chronological) age is the most appropriate criteria to restrict ART access, Cavaliere and Fletcher underline that specific age cutoffs make ‘all members of an age-group, who are homogenous in terms of similar birth years, […] homogenous in terms of imagined fertility, irrespective of *actual fertility*’.^57^ Although the authors prefer chronological age over lifestyle factors as a regulatory criterion to determine access to ART, they caution that ‘[chronological age discrimination] is not unproblematic’. Having an exclusively chronological age cutoffs for ART access risks to exclude people who, although being chronologically older, have similar (or higher) chances to conceive as compared with others who are chronologically younger. Indeed, the success of—for example—homologous IVF heavily depends on the aneuploidy rate, but also on the number of oocytes that can be retrieved after hormonal stimulation of the ovaries. This number, in turn, depends on the egg pool in the ovaries, which declines over time. This decline, however, varies considerably among individuals and thus both chronological age and the size of the ovarian pool of eggs influence the success rate of this ART.

### II.B. Biological Age(s)

An alternative conception of age is based on medico-biological elements, rather than on chronological ones. The idea of a biological conception of age stems from the observation that there can be significant variability among people of the same chronological age, thus calling for a measure of the aging process more sensitive to such individual differences.^58^ In this sense, it is possible to define the biological age of a person ‘as the individual’s present position with respect to his potential life span’.^59^ Indeed, from a biological perspective, humans are complex organisms comprised of various cells, tissues, and organs, each one with its own dynamic properties, and also ‘an openness to the environments inside and outside the human body’.^60^ Biological age is thus determined by the intrinsic biological characteristics of a person (eg, her genetic material), but also extrinsic elements that surround her, such as the time and place where she is born and raised. Indeed, the extensive literature on the social determinants of health—ie, ‘the conditions in which people are born, grow, work, live, and age’—has demonstrated the significant impact of socio-economic circumstances on health.^61^ This is because the biological aging of an organism (also called *senescing*) ‘results from a cumulative imbalance between damage and repair’, a balance that can be externally influenced by ‘reducing damage (by means of public-health efforts to enhance living conditions and to prevent disease, for example) and progress in increasing repair (by medical interventions, for example)’.^62^ The influence of external factors (such as a more advanced healthcare, but also better living conditions in some parts of the world) on the biological age is evident by the fact that in the last century the senescing of the human body in many countries has been postponed, so that ‘levels of mortality and other indices of health that used to prevail at age 70 now prevail at age 80, and levels that used to prevail at age 80 now prevail at age 90’.^63^ Biological aging can thus be measured not only by means of a calendar, but it requires the identification of biological markers that correlate with the process of senescence.^64^ In this respect, biological age refers specifically to the functionality of a certain organ(ism) as measured by specific biomarkers, whose value is affected by the passing of time, but also by other factors.

Biological age presents two main features. First, scientific advances and changing socio-economic conditions more broadly may potentially influence its progress. Although it is impossible to slow down chronological aging, affecting (and especially reducing) the biological age is the objective of many scientific efforts.^65^ This means that advances in science might have a substantial impact on the biological age of humans: for example, new technologies involving the transfer of mitochondria have been develop to allegedly rejuvenate the eggs of women when these are biologically old.^66^ Naturally, the implementation of such scientific advances depends on societal preferences. Moreover, the betterment of living conditions is dependent upon factors beyond one’s choice. As Overall underscores whilst discussing the biological aspects of aging ‘being old is determined largely by social forces that may or may not make good food available, healthy work possible, adequate medical care accessible, and “lifestyle” habits a matter of real choice’.^67^ But the fact remains that biological age is less ‘invariable’ than its chronological counterpart. Second, the biological age of distinct components of the human body can be different. Indeed, the assessment of biological age does not necessarily target an individual as a whole, but also one (or more) of its ‘vital or life-limiting organ systems’.^68^ Scientists studying biological age do not talk only of aging persons, but also of ‘aging cells’ or ‘aging tissues’.^69^ With reference to the human body, it is thus possible to speak of several biological ages, depending on which functional sub-unit of the body is considered. This latter feature of biological age bears particular importance in the context of ART, as it is illustrated in the next section.

#### II.B.1. Biological age(s) and access to ART

Biological and medical elements are often at the center of the (ethical) debate on ART and potential upper age limits for prospective parents. This is also due to the fact that for women, there is a discrepancy between the rapid loss of functionality of their eggs as compared with other organ systems, and to the fact that age-related fertility decline is much more pronounced in women than in men. In this context, both scientific literature and public discourse often refer to the ‘biological clock’, a term used from the 1970s onwards to indicate the existence of a process of reproductive aging in women, whereby biological factors are ‘separating women’s lives into the discrete time frames of menstruating/reproductive and menopausal/non-reproductive years’.^70^ Only more recently, the term has been used also in reference to men.^71^ The biological clock discourse raises several questions, since it intertwines ‘contingent and culturally specific social arrangements with what are perceived to be objective biological facts’^72^ and brings about a sort of culturalization of the biological,^73^ which is discussed below in II.B.3. In the next paragraphs, we focus instead on some of the more specific criteria proposed in the medical literature to map the biological age of men and women, with a focus on reproduction and fertility in general.

The Stages of Reproductive Aging Workshop (STRAW) proposed a taxonomy for the process of biological aging concerning reproduction for women.^74^ The rationale for creating the taxonomy was the observation that ‘women do not begin reproductive function (puberty) nor end it (menopause) at a particular chronological age’.^75^ In the view of STRAW ‘with the realization that chronological age is a very poor indicator, the purpose of a staging system would be the identification of where a given woman was in the process of reproductive aging’.^76^ Based on biological data and reliable tests, STRAW delineated a staging system, according to which each woman can be classified in respect to her biological (reproduction-related) age. This staging system for biological age includes: a reproductive stage (divided into early, peak and late), the stage of menopausal transition (early and late), and postmenopause (early and late). Although the authors naturally admit that these stages have a chronological duration, they also explicitly underline that ‘the age range and duration for each of these […] stages are variable’.^77^ This system was revised in 2011 (eg, by adding a few sub-stages), but it remained grounded on the same principles. Indeed, the authors of the update underlined that ‘The STRAW+10 model [name of the updated model] does not use [chronological] age as a criterion for determining reproductive staging’.^78^ They also remarked—as part of the principles on which the revised staging system is based—that it would rely on objective biological data, including menstrual cycle criteria and biomarkers, and that it would ‘use criteria that are independent of [chronological] age’.^79^

Attempts at defining criteria to assess the biological age of men—with a focus on their reproductive capability—have also been carried out. In 1999, Heinemann and colleagues developed a rating scale to evaluate on a bio-medical basis the process of aging in males. They created the Aging Males’ Symptoms (AMS) scale, specifying that ‘although the term “symptoms” is often used in association with diseases or other conditions, we also use it for complaints that develop in the course of aging which are not obviously caused by a certain disease (symptoms of aging)’.^80^ Their objective was to generate a medical scale for the aging process of males that would help understand, amongst other things, the phenomenon of ‘male climacteric’, which would correspond to menopause in women. Amongst the symptoms identified and analyzed in the AMS, a section concerned the reproductive or sexual aspects of male aging, which was found to correlate with the clinical diagnosis of a ‘male climacteric’ or andropause. On this topic, a review by Vermeulen argued that an ‘andropause’ may not exist *strictu sensu*, but the author also underlined several factors that can be defined as revealing an aging process in men in relation to reproduction.^81^ He highlighted that semen quality gradually declines with age and that there is also a progressive decline in testicular function characterized by lower level of androgens in the blood. Moreover, he also underlined that the decline in testosterone serum levels correlates with decreased libido and that such an androgen deficiency causes erectile dysfunction in a rising proportion of aging men. All in all, this points at factors that can determine the biological age of men with respect to reproduction in a different way as to their chronological age. As the production of spermatozoa continues at high levels throughout adult life, increased numbers of changes in the methylation pattern of the genetic material of the spermatozoa have been observed with rising age, which would explain the increased prevalence of neurodevelopmental abnormalities in the offspring of older men.^82^

A biological conception of age raises specific questions about when an age limit for accessing ART is at stake. For example, the task force for ethics and law of the European Society of Human Reproduction and Embryology (ESHRE) reflected on whether the biological profile of a prospective mother should influence the upper age limit for women in relation to ART access.^83^ The task force observed that there is preliminary evidence that smoking has an impact on biological age, in that smokers aged 20 have a comparable fertility to non-smokers aged 30. They then highlighted that, if this evidence is confirmed, there might be reason to have age limitations that take this biological factor into account. According to this line of thinking, the biological age of prospective parents might become more decisive to determine access to ART than their actual chronological age. It must be noted that elements such as the smoking status (or also the Body Mass Index) of a person are influenced by both by individual-behavioral factors, and also have other origins (eg, socio-economic conditions). But, in the framework of ART, the fact remains that they can be considered as essential elements of biological age, since they interplay with the biomedical functioning of the human body. Indeed, as highlighted above, the concept of biological age is strictly related to the physical human body, but it is the product of both internal (eg, genetic) and external (eg, behavioral or socio-economic) factors.

#### II.B.2. Biological age-limits for ART access in the law

At first sight, it would seem improbable to conceive legislation that sets an upper age limit for access to ART based on exclusively biological age—eg, due to the practical difficulties in assessing it. However, there are some examples of legal age-limits to ART access that are centered on biological age. A clear instance thereof is contained in Swiss law. Article 4 of the act regulating ART states that ‘ovum and embryo donation […] are prohibited’.^84^ Swiss legal commentators have remarked that the prohibition of oocyte donation constitutes an implicit age limit, in that it excludes women with reduced reproductive potential from the possibility to access ART, unless they have previously cryopreserved their own eggs.^85^ Based on our analysis, it is evident that such a rule can be described as a biological age limit: access to ART is not restricted based on the exact chronological age of the prospective mother, but rather on a biological process happening along the STRAW model. A further example is from Germany, where for residents covered by the private health insurance scheme (a substitutive scheme to social health insurance which covers around 11percent of the population^86^), reimbursement of ART procedures does not depend on the chronological age of the prospective parents, but it is based on the chances of success of the procedure itself. That is, reimbursement is not tied to a specific chronological age but rather to an individualized assessment of reproductive health (eg, level of basal follicle-stimulating hormone) and fertility, which may predict the chance of success of an ART procedure.^87^ More specifically, the requirement to obtain reimbursement is that the chances of a clinical pregnancy in the specific cases are at least 15 percent, according to a series of medical considerations by the treating team.^88^

#### II.B.3. Advantages and disadvantages of biological age-limits

There are both advantages and disadvantages to considering biological age as a parameter to set limits for ART access. On the one hand, biological age is a more individualized and personalized measure of the aging process. This is due to the fact that the *potential capacity* of having children is tied to biological factors, which are related to the reproductive organs of both men and women. For example, post-menopause has historically represented (before the development of ART) a biological barrier that prevents women to conceive. Similarly, we now know that there are biological factors (eg, reduction of testosterone level and semen quality) which gradually reduce the capacity of men to procreate. Given the importance of such biological factors, it has been argued that biological age is a better prediction than chronological age with respect to fertility.^89^ Biological fertility may not only predict the likelihood of successful treatment, but also correlate with the financial cost of a treatment—since it can impact how many attempts prospective parents make before obtaining a viable pregnancy. It could thus constitute a better criterion to regulate access to ART.

On the other hand, there are also some considerable disadvantages of relying on biological age limits. First, the very idea of defining a biological age limit as a condition to access ART leads to complications, because two different individuals and different organ systems are at play in reproduction. Indeed, the biological age of the two persons who are involved may differ greatly, and a couple could be *collectively* less likely to conceive just due to the biological age of one partner. Consider, for example, a couple where the woman is biologically young, but the male partner is biologically very old (eg, very poor semen quality). Moreover, for women who might be carrying the pregnancy (at least), two distinct organ systems (and their biological age) are involved: the ovaries and the uterus.^90^ Although menopause might be considered as a watershed in the biological aging of the ovaries, the same does not hold true as far as the uterus is concerned. In fact, as shown by the possibility to obtain a pregnancy through oocyte donation, ‘the uterus retains its receptivity to embryo implantation for a substantial period of time after the ovarian function decays, as long as sufficient exogenous hormonal support is provided’.^91^ It is only recently that the debate on biological aging for women has come to focus—as a synecdoche—on oocyte age, although the female body has arguably more than one biological age.^92^ For these reasons, one would have to decide *whose* biological age(s) could count as a parameter to limit ART access: the biological age of the male’s reproductive apparatus, the biological age of the female’s oocytes, and/or of her uterus?^93^ Very importantly, one would also have to consider the biological age of the prospective mother *as a whole*, eg, her capability of carrying the pregnancy to term without excessive complications both for her (these might include eg, hypertension, pre-eclampsia and diabetes) and for the baby (eg, those related to premature delivery).

Second, using biological age limits for regulating access to ART might seem at odd with the very objective of ART, which is indeed to circumvent biological boundaries of reproduction. Although it could be argued that ART should only be used to remedy pathological (eg, due to a disease) rather than physiological (eg, due to biological aging) infertility, drawing an exact line between the two is not always easy. Moreover, even if it were easy to determine, limiting the use of ART to those cases where infertility is caused by pathological factors might nevertheless be perceived as unjust. Indeed, the biological age of people (and their organs) might be influenced by an illness (eg, cancer) but also by poor lifestyle choices (eg, smoking) that are co-determined by their socio-economic status. If access to ART is restricted on such bases, this might risk to make people ‘personally and morally responsible for subfertility’.^94^ The biological aging of the human body can be slowed down by adopting a healthier lifestyle, but the latter is to a considerable extent socially determined. In consequence, limiting access to ART based on biological age could be considered unjust, since it would favor people with higher socio-economic background over those who are *biologically older* for no fault of their own. This point is especially relevant given that there are a few studies indicating that lower socio-economic status is already determining decreased access to ART^95^ and also decreased chances of success.^96^ Therefore, insisting too much on biological age might further exacerbate these existing inequities.

Third, biological considerations about reproductive age are particularly susceptible to being indirectly influenced by normative, political, and socio-cultural considerations, like the narrative of the ‘biological clock’ shows. As mentioned above, this term has been used in the past decades to refer to the existence of biological limits to reproductive time, especially for women. However, far from being grounded only on biological observations, it has turned into a heteronormative narrative ‘loaded with socially specific assumptions about how and under which conditions one should become a parent’.^97^ Indeed, ‘by intertwining contingent and culturally specific social arrangements with what are perceived to be objective biological facts’,^98^ the narrative of the biological clock commingles biomedical observations (about age-related fertility decline) with socio-cultural conceptions about appropriate reproductive time and with the chronological conception of time as linear and irreversible. The narrative of the biological clock corroborates the idea that female fertility is characterized by standardized and universal boundaries expressed in chronological terms, but based on allegedly objective biomedical considerations,^99^ which is ironic given that one crucial element of biological age would actually be that it is individualized and not necessarily in line with chronological age. Martin demonstrated how ideas on biological age (in particular through the narrative of the biological clock) are often implicitly conveyed through chronological age limits, which are, however, biologically justified.^100^ This can have a profound impact on how women perceive fertility. In her interview study, participants described age 35 as a perceived mystical ‘magic number’ and although they ‘are unsure about the medical significance of age 35’ they ‘still use bio-medicalized language to discuss 35 as a cutoff point or a risk calculator.’^101^ Biological age, also due to its complexity, thus risks to be translated into simple chronological age limits, which are often embedded into the narrative of the biological clock and thus entails an overlapping of biological issues with socio-cultural and normative conceptions about appropriate childbearing time.

Lastly, pure biological age limits raise questions of gender equality. If it is now technically possible to have children beyond menopause, why should the latter continue to be considered a meaningful biological age limit, as it is the case in Switzerland, for example? Even if menopause were to be regarded as a *physiological* sign of infertility, removing it could be considered ethically desirable as it would facilitate ‘the broader social goal of achieving reproductive equality between men and women’.^102^

### II.C. Socio-Cultural Age

The third alternative conception of age is based on socio-cultural perceptions about the presence of different stages in life. MacNicol explains that socio-cultural age brings together the identity assigned and the differentiation of status accorded to the norms and expected behaviors that prevail in a society at a given moment.^103^ Similar to the biological conception of age, socio-cultural age is mutable (the former due to technological advances and the socio-economic conditions where people live, the latter due to changing societal views about life stages). However, differently from biological age, its substratum does not lie in the biomedical constitution of a person or her body, but rather in the perceptions about aging of the societal and cultural context. As Baars highlighted, it is ‘socio-cultural narratives about aging that articulate […] what counts as “young”, “normal”, “old”, “very old”’.^104^ Indeed, in a given context, the social-cultural narratives are often in charge of determining what it means *to age*, and—importantly—‘these narratives are not just “stories”: they carry structural weight in the way markets are organized, political power is exercised, income and life chances are distributed’.^105^ The power of the expectations that are connected to the socio-cultural conception of age is emphatically expressed by Neugarten and colleagues at the incipit of a study in which they empirically investigated the force of age norms in American society. The authors explain that ‘there exists what might be called a prescriptive timetable for the ordering of major life events: a time in the life span when men and women are expected to marry, a time to raise children, a time to retire’.^106^ This conception of age is often described through the metaphor of a *social clock*, ie, the vision that ‘social norms dictate when certain life transitions should occur, and people accordingly strive to time their major life events to match societal expectations’.^107^

As socio-cultural age is tied to the perceptions and the narrative of a certain context, it has two main features. First, it may vary with time, since the views that a certain population holds about the different stages of life are influenced by a variety of factors, including also biotechnological advances and changes in the average life expectancy. Second, socio-cultural age varies in space. For children born in 2020, the average life expectancy in Switzerland is of 85.1 years for women and 81 years for men, whereas in Lesotho it was of 54.2 and 47.7 years for women and men, respectively, in 2019.^108^^,^^109^ Clearly, this staggering difference in life expectancy has an influence on what is considered to be *young* or *old* depending on where such considerations are made. But changes in life expectancy are not the only reason why socio-cultural age varies in time and space. Also normative and moral attitudes within the human population might differ considerable from country to country and time to time. Schroots and Birrren underline that ‘age-related roles may change under the influence of history-normative events’ and thus ‘society will change as new birth cohorts replace older ones’.^110^ Regardless of variety through time and space, a constant in socio-cultural conception of aging seems to be that there is a general division in at least three different life stages (with potential sub-stages): adult-to-be, adult, and once-were-adult.^111^

Despite being different from chronological age, it must be noted that socio-cultural age is often expressed in chronological terms. This is due to the fact that the majority of modern societies rely on chronological age to define and speak about many of the life stages of socio-cultural age (such as school age, voting age, working age, retirement age etc.).^112^ But this does not change the fact that chronological and socio-cultural age are not the same. Consider the distinction between working age and retirement age. The passage between the two is often determined by a chronological cutoff, if a state establishes a chronological age limit at which people can retire. But socio-cultural preferences might also establish that people can become pensioners—and thus transition from working age to retirement age—based on other factors than their chronological age (eg, the fact that they have accumulated enough pension funds or they have worked enough years).

#### II.C.1. Socio-cultural age and ART access

Socio-cultural elements and socio-cultural age are extremely important in the context of ART. This is because there is an intimate connection between socio-cultural views and reproduction. Indeed, as correctly observed by Göttlich ‘although fertility has a biological limit, social expectations may also limit its timing’.^113^ This is because childbearing has traditionally been considered throughout many societies as a significant component of specific life stages, thus often creating the assumption that there are periods in the life of a person where it is normal (in the sense of *common*, *usual*) to have children. This is influenced by expectations around the appropriate time for the succession of different genealogical generations within a certain family. As specified above, socio-cultural age is characterized by its continuously changing boundaries, since the public’s perceptions about the length of different life stages vary with time. This holds true especially with regards to the existence of socio-cultural age limits to (assisted) reproduction. Whilst commenting public perceptions about parental age limits for accessing ART, Shufaro and Schenker underline that such limits may change very quickly, since even ‘the current presently acceptable age (limitations~50 years of age) were considered adventurous 20 years ago’.^114^

A feature of socio-cultural age that is particularly relevant in relation to reproduction is that there are often marked differences in the perception of the appropriate timing for having children between men and women. Indeed, the public often believes that age limits to parenthood should be lower for women than for men.^115^ Biomedical and physiological considerations are certainly a co-cause of this, since the life span, during which women can have children without ART, is usually more limited than that of men. However, this is certainly not the only reason, especially since—thanks to the development of ART—the life span during which women can conceive has expanded from a biomedical point of view (eg, with the possibility of resorting to oocyte donation, menopause does not represent an insurmountable biological age barrier anymore). Other factors remain persisting drivers of the different sociocultural age limits for parenthood between men and women. As it has been noted, one of them is the widespread sociocultural assumption that fathers only play a marginal role in the upbringing of a child, and they can thus be older at the time of conception since ‘the father’s death or inability is not considered to be a major threat to the well-being of the child’.^116^ This may be the reason why, as Braverman notes, it is very common for the media to portray the achievement of fatherhood of men in their 70s as something to celebrate, whereas for women the same thing is widely considered an outrage—possibly also due to the fact that conceiving at such an advanced age through ART is considered abnormal/unnatural.^117^

Several studies have explored the impact of socio-cultural age, by investigating public views concerning parenthood in general and parenthood through ART more specifically.^118^ In an important project by Settersten and Hägestad on cultural age deadlines for life-course transitions (ie, the passage from a phase of life to another, such as leaving home or getting married), the authors found that the perceived age limit for entering parenthood was very similar for both parents, but that for completing childbearing the limit was 39 for women and 44 for men.^119^ Another study showed that 43 percent of the participants thought that women should stop having children before the age of 50, whereas only 32 percent believed that 50 should be the age limit for men.^120^ A further study explored the public views on social age deadlines for childbearing in the European context, revealing that such deadlines are perceived to be on average at 41.7 years for women and 47.3 for men.^121^ Interestingly, the study showed that almost 60 percent of respondents believed that women should not have children after the age of 40, whereas only 46 percent of them believed that the social deadline for childbearing in men ought to be at 45 years of age. Moreover, the authors demonstrated the existence of substantial variations by country: for example, the mean maternal age socio-cultural deadline indicated by respondents spanned from 39.3 years in Hungary to almost 44 in the neighboring Austria. A more recent study conducted in the United States with 1427 respondents indicated that preferences for upper age-limits for women supported 54 years of age as a cutoff, whereas for men the (relative) majority of participants indicated 64 as a desired cutoff. ^122^

#### II.C.2. Socio-cultural age-limits for ART access in the law

Although all regulatory age limits for ART are arguably influenced also by socio-cultural factors, it is difficult to find cases where a socio-cultural conception of age features directly^123^ in the legal requirements dictating which parents can access ART. Indeed, there is no regulation that limits access to ART based explicitly on societal views on life-stages and on the appropriate timing for parenthood. There are, however, age-related limits to ART access which are more prone to accommodate socio-cultural perceptions about the appropriate age to become parents. A potential example is the Italian law on ART, where article 5 states that ‘it is possible to access medically assisted reproduction only for adult couples […] in potentially fertile age’.^124^^,^^125^ This criterion refers to the concept of ‘fertile age’, which is arguably a biological age limit, since whether a couple is fertile depends primarily on their biomedical makeup. At the same time, the criterion is very open-ended, and it might thus also lead—in its concrete operationalization—to situations where socio-cultural preferences (of the doctors treating the single couple or of society in general) play a substantial role. This is also due to the fact that defining the exact boundaries of ‘fertile age’ in a biological sense is not easy. As highlighted by Bühler,^126^ most statistics referenced in medical articles that try to define ‘natural/biological fertile age’ are actually extrapolated by demographic studies on historical or non-contraceptive population (such as Heffner^127^, which refers data from Jane^128^ and Jane et al.^129^). These articles overlook that, in the apparently biological notion of ‘fertile age’ derived from historical demographic data, it is difficult to distinguish the ‘natural – the biological – from what is culturally and socially controlled – e.g. frequency of sexual intercourse, social taboos, migration, birth control practices and marriage patterns’.^130^ Bühler acknowledges that there are also studies which tried to isolate biological from socio-cultural factors in the identification of what ‘fertile age’ is, such as one which investigated the outcome of artificial insemination of women at different ages showing that the probability of conception falls rapidly after 31.^131^ But—as Bühler rightly points out—these studies took place in a specifically controlled experimental setting, which diverges drastically from normal conditions. These considerations are not meant to deny that fertility has a significant biological substratum, but they are rather meant to underscore that a legal criterion for ART access based on the concept of ‘potentially fertile age’—such as the Italian one—can be particularly prone to be influenced by socio-cultural factors, including ideas about the appropriate time for parenthood.

Also the former French law on ART that prospective parents ‘have to be in procreative age’^132^^,^^133^ to access ART. Whilst commenting this rule, Fragu argued that the concrete operationalization of this age limit depends certainly on biological, but also social consideration ‘that would fix an identical limit regardless of the person’s sex’.^134^ In August 2021, this potentially ambiguous rule was modified, turning this age limit that implied both biological and socio-cultural considerations into a predominantly chronological one. According to the updated law:

‘artificial insemination, the use of gametes or of germinal tissues gathered, collected or preserved for medical assistance to procreation […] as well as the transfer of embryos […] can be performed : 1) until the forty-fifth birthday of the woman, single or within a couple, who will carry the child; 2) until the sixtieth birthday of the member of the couple who will not carry the child.^135^

#### II.C.3. Advantages and disadvantages of socio-cultural age-limits

Limiting access to ART based on a socio-cultural conception of age like in Italian law can be useful in that it leaves single clinics a margin of appreciation to decide whether prospective parents should be granted access to ART. It could also be praised because, due to its open-endedness, a purely socio-cultural age limit caters for potential changes in public perception, without the need to update the law—which would be necessary, if the norms established a purely chronological age limit.

On the other hand, socio-cultural age limits can be subject to several criticisms. First, if they are expressed in way like the Italian case, they may be very indeterminate, thus generating *legal* uncertainty and—more importantly—uncertainties for the people who consider turning to ART. This could also open up the risk of exploitation by clinics who decide to interpret the limits very loosely to treat as many couples as possible, especially in case of wealthy prospective parents. Second, socio-cultural perceptions about life stages are often expressed in chronological terms, and they lead to delineating chronological age cutoffs for accessing ART, which are—however—based on socio-cultural assumptions, as outlined in II.A.3. This is problematic because an apparently chronological age limit can be actually be based on (and reinforce) public perceptions about age-deadlines. The latter are difficult to be empirically collected in an accurate fashion, and—even if it were simply to quantify what socio-cultural perceptions indicate—they are not necessarily *right* from a normative and moral perspective. In fact, in the field of empirical bioethics, it has long been clarified that majority opinions by the public cannot be used by itself to draw sound normative conclusions.^136^ Thus, legally limiting access to ART with chronological age cutoff based on socio-cultural conceptions about life stages could help perpetuate unjustified preconceptions about the role of men and women in respect to childrearing and their capacity to care for children. One could thus argue that access to ART should only be determined by prognostic factors relating to the success and safety of the procedure, rather than social perceptions about age deadlines. Third, liming access to ART based on socio-cultural views on what constitutes *childbearing age* leads to a catch-22 situation: the public might disapprove of parenting beyond a certain age exactly because (and until) that is banned. Indeed, empirical research has shown a positive correlation between accessibility to ART and later childbearing deadlines as perceived by the public.^137^

## III. PROPOSING A TEMPLATE FOR AGE LIMITS IN ART LAW

In the preceding sections, we have offered a taxonomy of the different meanings of age that are relevant for the legal context of ART. We have further illustrated a series of regulations containing age-limits for accessing ART that mirror the different conceptions of age, and we have discussed the advantages and disadvantages of using each of these conceptions in legislation. In this way, we have shown how important it is to understand ‘what kind of age we are talking about’ to accurately analyze the pros and cons of policies that limit access to ART based on how old parents are. Only grasping the distinct features that each conception of age possesses can provide the basis to start a discussion whether age-limits are appropriate in a certain context and time, how they can and should be formalized in legislation or guidelines, and whether they ought to be changed as technologies (and society) evolve.

But which conception of age should then be preferred in drawing limits for accessing ART in the law? Although it is difficult to formulate recommendations of a general nature that leave aside the context in which they would be applied, a few preliminary indications based on our analysis can be given.

### III.A. No Explicit Age Limit of Any Kind in Hard Law: A Viable Solution?

First, one could argue that, given the many disadvantages of age limits based on any conception of age, the law should do away with any limits that refer to age, no matter the form. This solution seems impractical for two reasons. On the one hand, even if they were absent in hard law, age-related considerations about limiting access to ART would remain present elsewhere, and thus applied by practitioners, but without the certainty that having them enshrined in the law can offer. This may be detrimental for prospective parents, who may think that they can rely on ART to try and have children given the absence of limits in the law, but then be confronted by clinics who decide to treat until a certain chronological, biological age, or socio-cultural age that would exclude them. So, explicit legal age limits can help to avoid arbitrary discriminatory treatment, since the role that age plays in reference to accessing ART would be laid out in clearer terms. On the other hand, having explicit age limits in the law may help to generate a debate on their appropriateness. The law has, especially in delicate bioethical matters, both an expressive and a communicative function, meaning that explicit legal rules ‘set the stage for further public debate and political deliberation’^138^ on the matter that they regulate. If the role that age (chronological, biological or socio-cultural) plays were to be determined by the individual clinic or healthcare professionals, it would be kept out of the public eye, and it would thus be more difficult to discuss whether any age cutoff is appropriate. A study in the US—where there are no age limits for ART in hard law—showed that clinicians consider parental age, but in a very uneven and anecdotal fashion, thus revealing some of the problematic issues of not having legal age limits.^139^

### III.B. Chronological Age Limits Adjusted by Biological Considerations

So, if the law then is to contain age limits, what conception of age should they embody? To answer that, one must first acknowledge that purely biological and purely socio-cultural age limits are much less widespread. This is because they can lead to situations that can be perceived as discriminatory (eg, by setting menopause as a moment beyond which ART cannot be accessed) or because they can be difficult to operationalize (eg, think of the open-endedness of limiting access to people ‘in their fertile age’). In those countries where ART is regulated, it is therefore much more common to have chronological age limits. As we noted above, even France—one of the few countries that had an age limit with some socio-cultural (as well as biological) elements—has recently switched to a chronological one. In fact, as noted earlier, chronological age limits have the considerable advantages of being straightforward, thus providing a very concrete and easily measurable yardstick—both for individuals who are considering to turn to ART and for healthcare professionals with whom they consult—to take decisions in respect to family planning. The legal certainty that they offer comes, however, at a price: an *exclusively* chronological age-limit struggles to account for individual variations and it could lead to excluding persons who—despite being chronologically older—might still be biologically very likely capable of sustaining a viable pregnancy, as compared with other people who are chronologically younger.

Is there a way to account for this trend towards implementing chronological age limits in legislation, whilst at the same time, mitigating the drawbacks of this conception of age? We believe that this can be done by combining a chronological age limit with some elements deriving from the biological conception of age, while taking into account societal and technological evolutions. More specifically, we recommend that policymakers continue to second the trend towards chronological age limits, but with two conditions.

First, chronological age limits should account for individual variability, one of the most important features deriving from the biological understanding of age. This could be done by setting rules which permit access to ART until a certain chronological age, but which allow for exceptions based on a series of biological parameters (eg, high probability of having a clinical pregnancy, combined with a prognosis that reasonably excludes life-threatening complications for mother and child during pregnancy) that are evaluated by the treating healthcare professionals. In this way, prospective parents who are above the chronological age limit, but are ‘biologically’ young(er) would not necessarily be excluded. Clearly, this adds a layer of complication in clinical decision-making. Establishing when it is advisable to undergo ART from a biological perspective is not straightforward, whereas an age limit that is exclusively chronological is simple to apply. Indeed, using biological markers as variables to determine whether a person is *too old* to receive treatment requires thinking about specific cutoffs values (in this case biological, rather than chronological) beyond which ART should not be used. It also requires treating staff and prospective parents to take on more decisional responsibility: there will be cases, for example, where medical examinations reveal that a woman requiring access to homologous IVF has a sufficient ovarian reserve, but her overall health condition would involve high obstetric risks. These borderline cases, however, would be difficult to decide not only with a *soft* chronological age limit which allows for exceptions based on biological parameters, but also if there were an *exclusively* chronological limit (eg, 50 years for women) and the person in question would be just below such limit (eg, 47) and present the same clinical features. Moreover, it is anyway part of clinical routine for all prospective parents who access ART to undergo certain medical examinations to measure specific biological parameters, since—regardless of legal limits—treating clinics are obliged to offer ART only if these are not futile or dangerous for patients. The law should thus align to this reality and, instead of setting a limit which is *exclusively* chronological, it should also set a number of evidence-based biological parameters, which would *exceptionally* allow prospective parents to access ART even beyond the *soft* chronological age cutoff. To avoid that the exception becomes the rule, the law could require a specific burden of proof for the cases where ART are offered beyond the *soft* chronological cutoff, including, for example, a second opinion by another independent clinic explaining why, in the single case, the prospective parents can receive access to ART despite being (slightly) over the chronological limit. In a report on fertility treatment and pregnancy with high risks, the Ethics Committee of the American Society for Reproductive Medicine already advised to obtain a second opinion in cases where there are biological factors that would suggest not to grant access to ART, to reduce the risk that the decision is not taken with bias.^140^ Similarly, in the case of age, requesting a second opinion by an independent clinic could be mandated to help ensure that there are no negative or positive biases that have influenced the decision to grant access to ART beyond the chronological age cutoff.

Second, chronological age limits should be set in a way that takes into account societal and technological evolution. As we argued whilst describing the socio-cultural conception of age, the mere fact that views about appropriate time-frames for becoming parents exist is not a good enough reason to determine that prospective parents should get access to ART only within such time-frames. However, analyzing socio-cultural preferences on life stages also sheds light on a valuable element of this understanding of age, namely its evolving and shifting nature. This does not mean that chronological age limits should change because the views of society towards appropriate time for parenthood evolve. It should however remind us that setting a chronological age limit—even if adjusted through the first condition we highlighted above—should not be considered as a permanent and definitive solution. Indeed, societal demographic features may change with time—eg, life expectancy could increase—and medical technology may advance. Both of these sets of factors bear on the assessment whether prospective parents should be given access to ART, and they can thus offer good reason to change the legally established age-limit after a certain time. Practically, this could be done by incorporating sunset clauses into legislation on ART. A sunset clause is ‘a provision that determines the termination of a statute, specific provision, programme, or agency, unless there is solid evidence that the latter should be renewed for another fixed period’ where ‘his termination or renewal should only occur after an ex post evaluation has taken place’.^141^ For our case, the restriction on ART access based on a certain chronological age (with the biological adjustment suggested above) could be covered by a sunset clause obliging the lawmaker to revise such age limits after a set amount of time (eg, 5 or 10 years). This would help ensure that the law does not promote the view that there is a clear and immutable time in the life of prospective parents (especially mothers), beyond which achieving pregnancy is necessarily undesirable. On the contrary, it would encourage legislators and society at large to periodically reconsider whether changes in access requirements are necessary, based on factors such as changing demographic structures or new technological or medical advances.

### III.C. Potential Objections

There are at least two potential objections to our proposal. First, that it still begs the question of how to avoid socio-cultural and biased factors from influencing decision making where the chronological age cutoff is set; second, allowing to go beyond the age limit based on biological consideration might be unjust, as favors prospective parents with a socio-economic advantage, as their biological age may be better.

As to the first objection, it is true that chronological age limits are empty shells and that non-chronological considerations are needed to decide where to put them. ^142^ The risk is high then, that questionable biological or biased socio-cultural considerations are used to justify a chronological age cutoff, as is the case with the German legal limit for social health insurance described in II.B.3. This is a risk, however, that can be significantly reduced by ensuring that the motivations of the chronological cutoff are made explicit and that they are contestable. Moreover, when sunset clause are in place, there is an in-built mechanism to allow for renegotiations at regular intervals. Exploring where exactly should this chronological age limit be placed (ie, at which chronological age) is beyond the scope of this article. But it would be important that such a decision is made based on transparent evidence-based arguments and not—as is often the case today—by approximations and *rule of thumb*.^143^

As to the second objection, we acknowledge that our proposal to allow for exceptions based on biological parameters may favor socio-economically advantaged prospective parents, as they may be *biologically younger* due to the healthier lifestyle and environment that a better socio-economic situation is often associated with. This is the reason why Cavaliere and Fletcher^144^ prefer to have purely chronological age limits to ART access rather than limits of a biological nature, especially if related to lifestyle choices. Although we share their concern, we still find it compelling to have an age limit that allows couples whose biological parameters and biological age are exceptionally good to have access to ART irrespective of their chronological age. Although a chronological age limit has the advantages we highlighted above, it still presents the great disadvantage of being too clear cut. Therefore, preventing prospective parents from accessing ART only because they are just above the chronological age cutoff, with no regard for their individual biological age would be too restrictive, even if their younger biological age were influenced by their more advantageous socio-economic status. Although it is crucial to address the social determinants of health (which influence the biological age of individuals), we do not believe that this could be done by denying access to ART to those prospective parents whose biological age would make access medically advisable, despite their chronological age. It is naturally crucial that the assessment of biological age is made without bias towards prospective parents whose biological age might be impacted by underlying diseases related to behavioral factors (such as smoking).^145^ But this is a concern for any medical assessment related to ART access, regardless of the existence of age limits and their nature. Moreover, it is also important that the biological parameters which are selected to determine the exception to the chronological age limit are evidence-based and transparent, for the same reasons we underscored above (clarity and contestability).

## IV. CONCLUSION

Defining parental age limits in ART regulation access is particularly difficult given the ambiguity and multifaceted meaning of age itself. In this article, we reviewed the different conceptions of age which underpin a variety of existing legal rules on ART access in the Global North. We then outlined the advantages and disadvantages of having a legal age limit for ART based on the different conceptions of age by referring to several examples of legislations. Finally, we proposed a suitable template for defining legal age limits for ART access with the following features: (i) the age limit should be of chronological nature, and the cutoff should be defined in a transparent and evidence-based manner; (ii) it should be exceptionally allowed to access ART also beyond such chronological age limit, in case the individualized biological age of the prospective parents is particularly favorable, and this is established in an unbiased manner with a second opinion by a different independent medical team; (iii) the chronological age limit should be periodically subject to renegotiation by means of a sunset clause, based on technological advances and changed societal circumstances (eg, extended lifespan). Although we expect our proposal to raise objections—some of which we have anticipated and addressed—it can prompt discussions about legal age limit for ART that are informed by the multifaceted nature of the concept of age. It thus provides a clear framework for academic debate and, more importantly, for policy discussions on the appropriate parental age limits in legislation on ART access.

## COMPETING INTERESTS

The authors declare no conflicts of interest.

## FUNDING

The writing of this manuscript has been permitted by the financial support provided by the Swiss National Science Foundation (Weave/Lead Agency funding program, grant number 10001AL_197415/1, project title ‘Family Building at Advanced Parental Age: An Interdisciplinary Approach’). The funder has no role in the drafting of this manuscript and the views expressed therein are those of the authors.

## Footnotes

1 Christian De Geyter, *Assisted Reproductive Medicine in Switzerland*, Swiss Med Wkly (2012), http://doi.emh.ch/smw.2012.13569 (accessed June 9, 2022).

2
*Id.*

3 The European IVF-Monitoring Consortium (EIM) for the European Society of Human Reproduction and Embryology (ESHRE) et al., *ART in Europe, 2017: Results Generated from European Registries by ESHRE*, 2021 Human Reproduction Open hoab026 (2021).

4 Fertility Europe, *European Atlas of Fertility Treatment Policies*, https://fertilityeurope.eu/european-atlas-of-fertility-treatment-policies/ (accessed June 7, 2021).

5 K. Berg Brigham, B. Cadier & K. Chevreul, *The Diversity of Regulation and Public Financing of IVF in Europe and its Impact on Utilization*, 28 Human Reproduction 666 (2013).

6 For the scope of this article, we will however not include surrogate motherhood.

7 Andrea Büchler & Karène Parizer, *Maternal Age in the Regulation of Reproductive Medicine – A Comparative Study*, 31 International Journal of Law, Policy and the Family 269 (2017); Berg Brigham, Cadier, and Chevreul, *supra* note 5.

8
Swedish National Council on Medical Ethics, *Report: Assisted reproduction – ethical aspects*, (2013), https://smer.se/en/2013/02/28/assisted-reproduction-ethical-aspects/ (accessed Aug. 11, 2022).

9 Judith Daar et al., *Oocyte or Embryo Donation to Women of Advanced Reproductive Age: An Ethics Committee Opinion*, 106 Fertility and Sterility e3 (2016). The reasons authors give for selecting the age of 55 is ‘the limited data regarding maternal and fetal safety, as well as concerns related to longevity and the need for adequate psychosocial supports for raising a child to adulthood’.

10 On the difficulty of establishing appropriate and consistent (with the objective of a certain policy) age cutoff in the law, see e.g., Govind Persad, *Reforming Age Cutoffs*, 56 U. Rich. L. Rev. 1007 (2021).

11 Linda J. Heffner, *Advanced Maternal Age — How Old Is Too Old?*, 351 N Engl J Med 1927 (2004).

12 I. Bray, David Gunnel & George Davey Smith, *Advanced Paternal Age: How Old is Too Old?*, 60 Journal of Epidemiology & Community Health 851 (2006).

13 Kieron Barclay & Mikko Myrskylä, *Advanced Maternal Age and Offspring Outcomes: Reproductive Aging and Counterbalancing Period Trends: Advanced Maternal Age and Offspring Outcomes*, 42 Population and Development Review 69 (2016).

14 David Carslake et al., *Associations of Parental Age with Health and Social Factors in Adult Offspring. Methodological Pitfalls and Possibilities*, 7 Sci Rep 45,278 (2017).

15 Vincent Couture et al., *The Other Face of Advanced Paternal Age: A Scoping Review of its Terminological, Social, Public Health, Psychological, Ethical and Regulatory Aspects*, 27 Human Reproduction Update 305 (2021).

16 Brittany Harrison et al., *Advanced Maternal Age: Ethical and Medical Considerations for Assisted Reproductive Technology*, 9 IJWH 561 (2017).

17 For a defense on the this view, see e.g., Hub A. E. Zwart, *The Moral Significance of our Biological Nature*, 1 Ethical Perspectives 71 (1994).

18 R. N. Butler, *Age-Ism: Another Form of Bigotry*, 9 The Gerontologist 243 (1969).

19 Büchler and Parizer, *supra* note 7. For a reflection on the feasibility of having no legal age limits, see III.A.

20 Thomas B.L. Kirkwood, *Understanding the Odd Science of Aging*, 120 Cell 437 (2005).

21 James E Birren & W. R. Cunningham, *Research on the Psychology of Aging: Principles, Concepts and Theory*, *in*Handbook of the psychology of aging 3 (James E. Birren & K. Warner Schaie eds., 2nd ed. 1985).

22 Alexander A. Boni-Saenz, *Legal Age*, 63 BCL Rev. 521 (2022).

23
*Id.*

24 Barbara Adam, *Time*, 23 Theory, Culture & Society 119 (2006); Carmen Leccardi, *Rethinking Social Time: Feminist Perspectives*, 5 Time & Society 169 (1996).

25 Lucy van de Wiel, *The Time of the Change: Menopause’s Medicalization and the Gender Politics of Aging*, 7 IJFAB: International Journal of Feminist Approaches to Bioethics 74 (2014).

26
Gail Wilson, Understanding Old Age: Critical and Global Perspectives (2000).

27 Boni-Saenz, *supra* note 22.

28
*Id.*

29 Johannes J.F. Schroots & James E. Birren, *Concepts of Time and Aging in Science*, *in*Handbook of the Psychology of Aging 45 (James E Birren & K. Warner Schaie eds., 1990), https://linkinghub.elsevier.com/retrieve/pii/B9780121012809500097 (accessed June 8, 2022).

30
*Id.*

31 K. Warner Schaie, *A General Model for the Study of Developmental Problems*, 64 Psychological Bulletin 92 (1965).

32 James E Birren, *Principles of Research on Aging*, *in*Handbook of Aging and the Individual 3 (James E. Birren ed., 1959).

33 Jan Baars, *Concepts of Time and Narrative Temporality in the Study of Aging*, 11 Journal of Aging Studies 283 (1997).

34 Susan Bewley, Melanie Davies & Peter Braude, *Which Career First?*, 331 BMJ 588 (2005).

35 Arthur Caplan & Pasquale Patrizio, *Are You Ever Too Old to Have a Baby? The Ethical Challenges of Older Women Using Infertility Services*, 28 Semin Reprod Med 281 (2010).

36
*Id.*

37
*Id.*

38 Robert L. Klitzman, *How Old Is Too Old? Challenges Faced by Clinicians Concerning Age Cutoffs for Patients Undergoing In Vitro Fertilization*, 106 Fertility and Sterility 216 (2016).

39
*Id.*

40 Carrie Friese, Gay Becker & Robert D. Nachtigall, *Rethinking the Biological Clock: Eleventh-Hour Moms, Miracle Moms and Meanings of Age-Related Infertility*, 63 Social Science & Medicine 1550 (2006).

41 C Calhaz-Jorge et al., *Survey on ART and IUI: Legislation, Regulation, Funding and Registries in European Countries*, 2020 Human Reproduction Open hoz044 (2020).

42 Chronological age limits are lower, if the mother wishes to have the costs for the treatment covered by social health insurance.

43 Guido Pennings, *Belgian Law on Medically Assisted Reproduction and the Disposition of Supernumerary Embryos and Gametes*, 14 Eur J Health Law 251 (2007).

44 Calhaz-Jorge et al., *supra* note 41.

45
Federal Act on Medically Assisted Reproduction, (1998), https://www.fedlex.admin.ch/eli/cc/2000/554/en (accessed June 8, 2022).

46 See II.B.2 on a further age-related limit contained in Swiss legislation.

47 Klitzman, *supra* note 38.

48 Persad, *supra* note 10.

49 Boni-Saenz, *supra* note 22.

50 Anna Smajdor, *The Ethics of IVF Over 40*, 69 Maturitas 37 (2011).

51 § 27a para 3 Sozialgesetzbuch Fünftes Buch (SGB V), https://www.sozialgesetzbuch-sgb.de/sgbv/27a.html (accessed Aug. 12, 2022).

52 Federal Social Court (Bundessozialgericht - BSG), decision of 3rd March 2009 - B 1 KR 12/08 R.

53
German IVF Registry, *Yearly Report 2006*, https://www.deutsches-ivf-register.de/perch/resources/downloads/dirjahrbuch2006.pdf (accessed Aug. 12, 2022).

54
German IVF Registry, *Yearly Report 2020*, https://www.deutsches-ivf-register.de/perch/resources/dirjb2020en.pdf (accessed Aug. 12, 2022).

55 On the risk that biological considerations can hide socio-cultural assumptions about life stages, see also section 2.2.2.

56 Pennings, *supra* note 43.

57 Giulia Cavaliere & James Rupert Fletcher, *Age-Discriminated IVF Access and Evidence-based Ageism: Is There a Better Way?*, Science, Technology, & Human Values 016224392110219 (2021). Italics added.

58 Schroots and Birren, *supra* note 29.

59 Birren and Cunningham, *supra* note 21.

60 Jan Baars, *Concepts of Time in Age and Aging*, *in*The Palgrave Handbook of the Philosophy of Aging 69 (Geoffrey Scarre ed., 2016), http://link.springer.com/10.1057/978-1-137-39356-2_5 (accessed June 7, 2022).

61 Michael Marmot et al., *Closing the Gap in a Generation: Health Equity through Action on the Social Determinants of Health*, 372 The Lancet 1661 (2008).

62 James W. Vaupel, *Biodemography of Human Ageing*, 464 Nature 536 (2010).

63
*Id.*

64 Schroots and Birren, *supra* note 29.

65 C. Farrelly, *Has the Time Come to Take on Time Itself?*, 337 BMJ a414 (2008).

66 Nolwenn Bühler & Cathy Herbrand, *Powering Life through MitoTechnologies: Exploring the Bio-Objectification of Mitochondria in Reproduction*, 17 BioSocieties 99 (2022).

67 Christine Overall, *How Old Is Old? Changing Conceptions of Old Age*, *in*The Palgrave Handbook of the Philosophy of Aging 13 (Geoffrey Scarre ed., 2016), http://link.springer.com/10.1057/978-1-137-39356-2_2.

68 Birren and Cunningham, *supra* note 21.

69 Jan Baars, *Human Aging, Finite Lives and the Idealization of Clocks*, 18 Biogerontology 285 (2017).

70 Friese, Becker, and Nachtigall, *supra* note 40.

71 Sarah M. Lambert, Puneet Masson & Harry Fisch, *The Male Biological Clock*, 24 World J Urol 611 (2006).

72 Merav Amir, *Bio-Temporality and Social Regulation: The Emergence of the Biological Clock*, Polygraph 47 (2006).

73 Nolwenn Bühler, *The ‘good’ of Extending Fertility: Ontology and Moral Reasoning in a Biotemporal Regime of Reproduction*, 44 HPLS 21 (2022).

74 This was organized by the American Society for Reproductive Medicine, the National Institute on Aging, the National Institute of Child Health and Human Development, and the North American Menopause Society. Their purpose was not to address directly the topic of ART, but it is still relevant in this respect, as their taxonomy concerns biological age in respect to reproduction.

75 M. R. Soules et al., *Executive Summary: Stages of Reproductive Aging Workshop (STRAW)*, 4 Climacteric 267 (2001).

76
*Id.*

77
*Id.*

78 Siobán D. Harlow et al., *Executive Summary of the Stages of Reproductive Aging Workshop + 10: Addressing the Unfinished Agenda of Staging Reproductive Aging*, 15 Climacteric 105 (2012).

79
*Id.*

80 L. A. J. Heinemann et al., *A New ‘Aging Males’ Symptoms’ Rating Scale*, 2 The Aging Male 105 (1999).

81 A. Vermeulen, *Andropause*, 34 Maturitas 5 (2000).

82 Mingju Cao et al., *High-Resolution Analyses of Human Sperm Dynamic Methylome Reveal Thousands of Novel Age-Related Epigenetic Alterations*, 12 Clin Epigenet 192 (2020).

83 ESHRE Task Force on Ethics and Law, including et al., *Lifestyle-Related Factors and Access to Medically Assisted Reproduction*, 25 Human Reproduction 578 (2010).

84 Reproductive Medicine Act, RMA, *supra* note 45.

85 Andrea Büchler & Sandro Clausen, *Art. 3 Kindeswohl /IV. - VII.*, *in*Fortpflanzungsmedizingesetz (FMedG) 212 (Andrea Büchler & Bernhard Rütsche eds., 2020).

86
Miriam Blümel et al., Germany: Health system review (2020), https://apps.who.int/iris/handle/10665/341674.

87 As established in the Federal Court of Justice (Bundesgerichtshof - BGH), decision of 21st September 2005 - IV ZR 113/04, http://juris.bundesgerichtshof.de/cgi-bin/rechtsprechung/document.py?Gericht=bgh&Art=en&nr=34148&pos=0&anz=1 (accessed June 8, 2022).

88 Kyrill Makoski, *Recht der Reproduktionsmedizin*, *in*Münchener Anwaltshandbuch Medizinrecht (Tilman Clausen & Jörn Schroeder-Printzen eds., 3rd ed. 2020).

89 Basil C. Tarlatzis & Leonidas Zepiridis, *Perimenopausal Conception*, 997 Annals of the New York Academy of Sciences 93 (2003).

90 S.M. Nelson, E.E. Telfer & R.A. Anderson, *The Ageing Ovary and Uterus: New Biological Insights*, 19 Human Reproduction Update 67 (2013).

91 Yoel Shufaro & Joseph G Schenker, *Pregnancies beyond the Human Biological Fecundity*, 8 Womens Health (Lond Engl) 49 (2012).

92 Nolwenn Bühler, *The Making of ‘Old Eggs’: The Science of Reproductive Ageing between Fertility and Anti-Ageing Technologies*, 14 Reproductive Biomedicine & Society Online 169 (2022).

93 Needless to say, it would also depend on the type of ART at stake.

94 Cavaliere and Fletcher, *supra* note 58.

95 Katie Harris et al., *Socio-Economic Disparities in Access to Assisted Reproductive Technologies in Australia*, 33 Reproductive BioMedicine Online 575 (2016).

96 James F. Smith et al., *Socioeconomic Disparities in the Use and Success of Fertility Treatments: Analysis of Data from a Prospective Cohort in the United States*, 96 Fertility and Sterility 95 (2011).

97 Amir, *supra* note 74.

98
*Id.*

99 Martina Yopo Díaz, *The Biological Clock: Age, Risk, and the Biopolitics of Reproductive Time*, 84 Sex Roles 765 (2021).

100 See also our discussion in 2.1.3.

101 Lauren Jade Martin, *Pushing for the Perfect Time: Social and Biological Fertility*, 62 Women’s Studies International Forum 91 (2017).

102 Merryn Elizabeth Ekberg, *Assisted Reproduction for Postmenopausal Women*, 17 Human Fertility 223 (2014).

103 John MacNicol, *Âgisme et Discrimination Dans le Monde du Travail. Regard Historique Sur le Cas Britannique*, 59 Mouvements 24 (2009).

104 Jan Baars, *Time and Aging: Enduring and Emerging Issues*, *in*International Handbook of Social Gerontology 367 (Dale Dannefer & Chris Phillipson eds., 2010).

105
*Id.*

106 Bernice L. Neugarten, Joan W. Moore & John C. Lowe, *Age Norms, Age Constraints, and Adult Socialization*, 70 American Journal of Sociology 710 (1965).

107 Karen S. Rook, Ralph Catalano & David Dooley, *The Timing of Major Life Events: Effects of Departing from the Social Clock*, 17 American Journal of Community Psychology 233 (1989).

108 Swiss Federal Statistical Office, *Life expectancy*, https://www.bfs.admin.ch/bfs/en/home/statistics/population/births-deaths/life-expectancy.html (accessed June 8, 2022).

109 World Health Organisation - Global Health Observatory Data Repository (African Region), *Life expectancy and Healthy life expectancy - Data by country*, https://apps.who.int/gho/data/view.main-afro.SDG2016LEXv?lang=en (accessed June 8, 2022).

110 Schroots and Birren, *supra* note 29.

111 Andreas Göttlich, *‘When I Was Young’ The Idealization of the Interchangeability of Phases of Life*, 36 Hum Stud 217 (2013).

112 Juliette Rennes, *Âge Biologique Versus Âge Social : Une Distinction Problématique:*, n° 117 Genèses 109 (2019).

113 Jiřina Kocourková et al., *How Old is Too Old? A Contribution to the Discussion on Age Limits for Assisted Reproduction Technique Access*, 30 Reproductive BioMedicine Online 482 (2015).

114 Shufaro and Schenker, *supra* note 93.

115 Ekberg, *supra* note 104.

116 G. Pennings, *Age and Assisted Reproduction*, 14 Med Law 531 (1995).

117 Andrea Mechanick Braverman, *Old, Older and Too Old: Age Limits for Medically Assisted Fatherhood?*, 107 Fertility and Sterility 329 (2017).

118 It is important to note that these studies investigated perceptions about different life stages by referring to chronological age limits. This is due to the fact that—as noted above in II.C—in modern societies sociocultural age is normally expressed in chronological terms. But this does not change the fact that—despite asking about *chronological* cutoffs—the studies where investigating socio-cultural expectations about age.

119 R. A. Settersten & G. O. Hagestad, *What’s the Latest? Cultural Age Deadlines for Family Transitions*, 36 The Gerontologist 178 (1996).

120 M. C. Bowman & D. M. Saunders, *Community Attitudes to Maternal Age and Pregnancy after Assisted Reproductive Technology: Too Old at 50 Years?*, 9 Human Reproduction 167 (1994).

121 F. C. Billari et al., *Social Age Deadlines for the Childbearing of Women and Men*, 26 Human Reproduction 616 (2011).

122 Malinda S. Lee et al., *Public Perspectives on Placing Age Limits on Men and Women Seeking Fertility Treatment*, 111 Fertility and Sterility 497 (2019).

123 As we outlined above at the beginning of II, II.A.3 and II.B.3, often socio-cultural considerations are indirectly used to justify chronological age cutoff or they are mixed with biological considerations in the biological clock narrative. Consider, for example, the Swiss rule discussed in II.B.2: the fact that egg donation is not allowed, whereas sperm donation is permitted certainly shows the impact of socio-cultural gender norms.

124
Legge 19 febbraio 2004, n. 40 - Norme in materia di procreazione medicalmente assistita., https://www.trovanorme.salute.gov.it/norme/dettaglioAtto?id=4538&completo=true (accessed June 8, 2022). Original in Italian, our translation.

125 For the reimbursement of most ART interventions by the Italian National Healthcare Service, there is a maximum chronological age limit of 46 years at the national level. See Decreto Del Presidente Del Consiglio Dei Ministri 12 Gennaio 2017, https://www.gazzettaufficiale.it/eli/id/2017/03/18/17A02015/sg (accessed June 8, 2022).

126
Nolwenn Bühler, When Reproduction Meets Ageing: The Science and Medicine of The Fertility Decline (1st ed. 2021).

127 Heffner, *supra* note 11.

128 Jane Menken, *Age and Fertility: How Late Can You Wait?*, 22 Demography 469 (1985).

129 Jane Menken, James Trussell & Ulla Larsen, *Age and Infertility*, 233 Science 1389 (1986).

130
Bühler, *supra* note 128.

131 B M van Noord-Zaadstra et al., *Delaying Childbearing: Effect of Age on Fecundity and Outcome of Pregnancy*, 302 BMJ 1361 (1991).

132 Article L2141–2 of the Code de la santé publique - Version en vigueur au 01 janvier 2021, https://www.legifrance.gouv.fr/codes/id/LEGIARTI000038310837/2021-01-01/ (accessed June 8, 2022).

133 Berg Brigham, Cadier, and Chevreul, *supra* note 5.

134 Estelle Fragu, *La Reforme Française de la Bioéthique: Vers un Nouveau Modèle de Reproduction ?*, URPP Human Reproduction Reloaded | H2R (University of Zurich). Working Paper Series (2022). Original in French, our translation.

135
Décret n° 2021–1243 du 28 septembre 2021 fixant les conditions d’organisation et de prise en charge des parcours d’assistance médicale à la procréation, https://www.legifrance.gouv.fr/jorf/id/JORFTEXT000044111531 (accessed June 8, 2022). Original in French, our translation.

136 Daniel P. Sulmasy & Jeremy Sugarman, *The Many Methods of Medical Ethics (Or, Thirteen Ways of Looking at a Blackbird)*, *in*Methods in Medical Ethics 3 (Jeremy Sugarman & Daniel P. Sulmasy eds., 2001).

137 Billari et al., *supra* note 123.

138 B. C. Van Beers, *Is Europe ‘Giving in to Baby Markets?’ Reproductive Tourism in Europe and the Gradual Erosion of Existing Legal Limits to Reproductive Markets*, 23 Medical Law Review 103 (2015).

139 Klitzman, *supra* note 38. In the same study, clinicians simultaneously call for more guidance, but oppose the introduction of age cutoffs in the law. This might be because they genuinely think that governmental intervention would complicate things, or because they are generally averse to the law-ification of medical practice or because they feel that legal cutoffs would limit the exercise of their medical discretion. However, even if these motivations were valid, they may not be enough to outweigh the clarity that legal age limits would entail for patients trying to access ART. Moreover, reducing medical discretion in such a practice might be beneficial, given how prone clinicians might be to bias in decision making concerning reproduction.

140 Judith Daar et al., *Provision of Fertility Services for Women at Increased Risk of Complications during Fertility Treatment or Pregnancy: An Ethics Committee Opinion*, 106 Fertility and Sterility 1319 (2016).

141 S. Ranchordas, *Sunset Clauses and Experimental Regulations: Blessing or Curse for Legal Certainty?*, 36 Statute Law Review 28 (2015).

142 See also Section 2.1.2.

143 For a discussion on how chronological age cutoff in the law are sometimes picked quite superficially, see the reflection on age cutoff for Medicare and Social Security in Persad, *supra* note 10.

144 Cavaliere and Fletcher, *supra* note 58.

145 Daar et al., *supra* note 142.

